# OP79 Experience Report: Strengthening Health Technology Assessment Through The Brazilian Coalition For Evidence Meeting

**DOI:** 10.1017/S0266462325101335

**Published:** 2025-12-29

**Authors:** Ana Carolina Nonato, Jessica Farias Dantas Medeiros, Maíra Barroso Pereira, Luis Phillipe Nagem Lopes, Ana Lígia Passos Meira, Luciane Lopes

## Abstract

**Introduction:**

Health technology assessment (HTA) events are essential for fostering collaboration among stakeholders, enhancing methodologies, and addressing challenges in evidence integration. These events are particularly valuable for HTA centers (HTACs), which play a crucial role in evidence-informed policymaking in Brazil. This report highlights the experiences and outcomes of the first in-person meeting of the Brazilian Coalition for Evidence (CBE).

**Methods:**

The CBE is a network comprising over 40 institutions dedicated to evidence-informed social interventions. The meeting was held on 8 to 9 August 2024, at the University of Sorocaba, Brazil, in conjunction with the seventh International Workshop on Rational Use of Medicines and the fourth Worknowledge in Evidence-Informed Policies. The agenda featured thematic panels on integrating evidence ecosystems and the role of qualitative evidence in HTA. The target audience included representatives from academia, public administration, civil society, HTACs, and evidence centers, fostering cross-sectoral dialogue and capacity building.

**Results:**

Approximately 100 participants attended the meeting, sharing insights on HTA’s critical role in advancing evidence-informed policies in Brazil and Latin America. Key sessions included the plenary, which explored the integration of qualitative perspectives into HTA frameworks. Presentations from the Evidence-Informed Policy Network – Americas (EVIPNet Americas), Health Technology Assessment Network of the Americas (RedETSA), and the Brazilian Health Technology Assessment Network (REBRATS) showcased successful regional HTA initiatives, highlighting their impact on local governance and decision-making. Discussions underscored the need to expand HTA applications, addressing social challenges and emphasizing relevance to sustainable development.

**Conclusions:**

The meeting highlighted HTA’s potential to transform political landscapes by connecting science with decision-making. By equipping stakeholders with advanced tools and fostering cross-sectoral collaboration, it laid a foundation for more equitable and effective public policies. These efforts underscore the importance of investment in evidence ecosystems for long-term societal benefits, and highlight the significance of collaborative networks such as CBE, REBRATS, and EVIPNet.

